# Absence of Bacteria on Coronary Angioplasty Balloons from Unselected Patients: Results with Use of a High Sensitivity Polymerase Chain Reaction Assay

**DOI:** 10.1371/journal.pone.0145657

**Published:** 2015-12-22

**Authors:** Gorm Mørk Hansen, Martin Nilsson, Claus Henrik Nielsen, Palle Holmstrup, Steffen Helqvist, Tim Tolker-Nielsen, Michael Givskov, Peter Riis Hansen

**Affiliations:** 1 Department of Cardiology, Gentofte University Hospital, Hellerup, Denmark; 2 Costerton Biofilm Center, Department of Immunology and Microbiology, Faculty of Health and Medical Sciences, Copenhagen University, Copenhagen, Denmark; 3 Institute for Inflammation Research, Department of Infectious Diseases and Rheumatology, Rigshospitalet, Copenhagen, Denmark; 4 Section of Periodontology, Department of Odontology, Faculty of Health and Medical Sciences, Copenhagen University, Copenhagen, Denmark; 5 Department of Cardiology, Rigshospitalet, University of Copenhagen, Copenhagen, Denmark; 6 Singapore Center on Environmental Life Sciences Engineering (SCELSE), Nanyang Technological University, Singapore; Medical University of South Carolina, UNITED STATES

## Abstract

Periodontitis is a chronic, bacterially-induced inflammatory disease of the tooth-supporting tissues, which may result in transient bacteremia and a systemic inflammatory response. Periodontitis is associated with coronary artery disease independently of established cardiovascular risk factors, and translocation of bacteria from the oral cavity to the coronary arteries may play a role in the development of coronary artery disease. Very few studies have used angioplasty balloons for *in vivo* sampling from diseased coronary arteries, and with varying results. Therefore, the aim of this study was to assess if bacterial DNA from primarily oral bacteria could be detected on coronary angioplasty balloons by use of an optimized sampling process combined with an internally validated sensitive polymerase chain reaction (PCR) assay. Coronary angioplasty balloons and control samples from a total of 45 unselected patients with stable angina, unstable angina/non-ST elevation myocardial infarction, and ST-elevation myocardial infarction (n = 15 in each group) were collected and analyzed using a PCR assay with high sensitivity and specificity for 16S rRNA genes of the oral microbiome. Despite elimination of extraction and purification steps, and demonstration of sensitivity levels of 25–125 colony forming units (CFU), we did not detect bacterial DNA from any of the coronary angioplasty balloons. A subsequent questionnaire indicated that the prevalence of periodontitis in the study cohort was at least 39.5%. Although coronary angioplasty balloons are unlikely to be useful for detection of bacteria with current PCR techniques in unselected patients with coronary artery disease, more studies are warranted to determine the extent to which bacteria contribute to atherosclerosis and its clinical manifestations and whether the presence of bacteria in the arteries is a transient phenomenon.

## Introduction

Cardiovascular disease (CVD) is the leading cause of death worldwide and its primary cause, atherosclerosis, is a chronic inflammatory disease of the arteries, which is strongly associated with risk factors such as hypertension, smoking, diabetes, obesity, hyperlipidemia, a family history of CVD, and socioeconomic deprivation. However, for more than three decades it has been hypothesized that infection may play a role in the atherosclerotic process from initial endothelial dysfunction to clinical manifestations, e.g., myocardial infarction (MI) and stroke [1–8]. Previous research has largely been directed at single pathogen models for the association between infection and atherosclerosis, and some of the usual suspects have included *Chlamydophilia pneumonia*, *Helicobacter pylori*, *Porphyromonas gingivalis* and *Cytomegalovirus* (*CMV*) [6,9–11]. However, more contemporary studies have been orientated towards polymicrobial models of the association between infection and CVD, reflecting the view that the total pathogenic infectious burden in any individual is more important than any singular microbe as a risk factor for CVD [6–8,12].

Periodontitis, occurring in as much as 50% of the population over 50 years of age, is a polymicrobially-induced chronic inflammatory disease in which multiple bacterial species, including anaerobic Gram negative rods and spirochetes organized in a biofilm, colonize the periodontal pockets [13]. The resulting inflammatory reaction degrades the tooth supporting connective tissue and bone, and may lead to a systemic inflammatory response [4,14,15]. Though a causal relationship is uncertain, a growing body of observational, interventional, and mechanistic studies have associated periodontitis with CVD, largely independent of overlapping risk factors such as smoking, diabetes and socioeconomic factors [1,2,16–21]. Indeed, there have been numerous reports on detection of bacterial DNA in atherosclerotic tissue samples removed at surgery or autopsy as well as catheter-based directional coronary atherectomy, and in intracoronary thrombi aspirated during primary percutaneous coronary intervention (PCI), respectively, including a few reports indicating the presence of viable microorganisms [22–28]. However, several large randomized controlled studies with antibiotic treatment have failed to demonstrate improved cardiovascular outcomes and mortality in patients with coronary artery disease, and more recently it has been suggested that organization of bacteria in antibiotic-resistant biofilms may have contributed to these negative results [29–34].

The usefulness of coronary angioplasty balloons for retrieval of atherosclerotic tissue material for detection of bacteria has previously received very limited interest and results have been both positive and negative [35–37]. During PCI, the coronary angioplasty balloon at the catheter tip is inflated and pressed against the diseased atherosclerotic vessel wall under high (usually 10–20 atm) pressure and it is hypothesized that folds in the deflated angioplasty balloon may contain representative tissue material including bacterial DNA, which can then be amplified by use of polymerase chain reaction (PCR) assays. In these prior studies, techniques were either aimed at detection of specific microbes, or a broad spectrum of bacteria were targeted using standard environmental ‘universal’ 16S RNA primers, which anneal to the highly conserved regions of the bacterial 16S rRNA gene [35–37]. However, since no truly universal bacterial primers exist, the specific choice of ‘universal’ primers has a significant impact on how the microbiome is characterized upon amplification and sequencing [38–43]. Furthermore, manipulation of sampled material from angioplasty balloons through steps with washing buffers, and extraction and purification techniques, respectively, could result in contamination and/or loss of material and lead to misrepresentation of the original sample microbiome once sequenced. In addition, previous studies reported combined results from both patients with stable angina and those with acute coronary syndromes and included either unselected patients or subjects with periodontitis [35–37]. Also, these studies failed to use arterial blood as a negative control to exclude that positive samples represented circulating blood rather than atherosclerotic tissue material retrieved from the diseased coronary artery segment by the angioplasty balloon. Therefore, we devised a method with minimal manipulation of the original sample by elimination of washing, extraction, and purification steps, and by use of arterial blood controls, respectively. Furthermore, our method was designed to achieve high sensitivity and specificity by means of carefully selected 16S RNA primers aimed at the oral microbiota. With use of this optimized sampling process and internally validated PCR assay we hypothesized that: 1) bacteria primarily originating from the oral microbiome could be sampled *in vivo* by use of angioplasty balloons from unselected patients undergoing PCI, 2) the severity of the clinical manifestations of coronary atherosclerosis, i.e., stable angina, unstable angina (UAP), non ST-segment elevation MI (NSTEMI), or ST-segment elevation MI (STEMI) in these patients was proportional to the amount and diversity of the bacteria detected on their respective angioplasty balloons.

## Materials and Methods

### Patient population and sample retrieval

A total of 45 patients subjected to PCI of a *de novo* coronary lesion were enrolled at two tertiary invasive cardiology centers (Gentofte University Hospital and Copenhagen University Hospital Rigshospitalet), including 15 patients with stable angina, 15 patients with UAP or NSTEMI, and 15 patients with STEMI. Patients with a history of infection requiring systemic antibiotic treatment and those with immunocompromising conditions, e.g., chemotherapy or chronic inflammatory diseases receiving immunosuppressive treatment, within a three months period prior to admission were excluded from the study as were subjects with clinical and biochemical signs of active infection. After a mean 17 months after PCI, two patients had died and the remaining patients were contacted by phone and subjected to a standardized questionnaire to determine if at the time of PCI they had periodontitis diagnosed by a dentist, the number of remaining teeth, the number of loose teeth, and whether gum bleeding occurred regularly when performing oral hygiene. The study was approved by The Regional Committee on Health Research Ethics of The Capital Region of Denmark (protocol number H-2-2012-154) and written, informed consent was given by all participating patients.

Immediately upon withdrawal of the angioplasty catheter from the guiding catheter, the distal balloon part of the catheter (approximately 20 mm in length) was cut directly into sterile, RNAse/DNAase-free tubes, sealed, labeled and quickly frozen at -80°C. Only angioplasty balloons used for predilation were sampled and subsequent balloons used for drug-eluting stent implantation in individual patients were specifically avoided, due to their polymeric coatings and cytostatic drug release which could potentially interfere with bacteria and the PCR assay. As negative controls we used a 20 mm control segment of the respective angioplasty catheter taken 5–7 cm proximally from the balloon. This part of the catheter was in contact with the arterial blood and potentially (in cases of more distal coronary artery dilatation) in passive contact with the arterial wall of the proximal coronary artery segments. If the electro-chemical properties of angioplasty catheter material promoted passive adherence of circulating bacteria from the blood (and no additional bacteria were specifically retrieved from the dilated coronary artery segment) these catheter control segments were expected to be positive to the same extent as the angioplasty balloons. In addition, to control for positive samples being the result of contamination from arterial blood, blood samples were drawn from the femoral artery sheath and transferred into heparinized collection tubes. All sample collections were done using single use sterile equipment and samples and controls were stored at -80° C until analysis.

### PCR assay and internal validation

The collected samples were thawed on ice in random order, and the angioplasty balloons and catheter control segments were cut in smaller pieces, allowing them to fit directly into a pre-prepared PCR master mix in standard 200 μL PCR strips. Five microliter of the arterial blood sample was likewise pipetted directly into prepared PCR tubes. Measures to minimize risk of contamination during analysis were carefully undertaken, and all handling was done in laminar flow cabinet using sterile equipment. PCR master mix, flow cabinet, and equipment were UV-radiated for 10–20 min prior to use, to reduce risk of DNA contamination. PCR was done using Thermo Scientific Phusion Blood Direct PCR Kit (Thermo Fisher Scientific, Waltham, Massachusetts, USA). To allow for identification of a broad spectrum of bacteria we used primers annealing to the highly conserved regions of the bacterial 16S rRNA gene. The forward primer was 357F (5’-TCC TAC GGG AGG CAG C-3’) and reverse primer was 1391R (5’-GAC GGG CGG TGT GTR CA-3’) generating a 1034 bp product (using the 16S rRNA gene of *Escherichia coli* as reference). This specific choice of primer pair was based on matching the sequences of several combinations of commonly used ‘universal’ 16S RNA primers with the online 16S RNA library of the Human Oral Microbiota Database (HOMD) [44]. This was done in order to achieve 100% annealing match with 16S rRNA gene sequences of as many oral species as possible and with the chosen primer pair this was achieved with 95% of the >800 species sequenced and available in HOMD. Among these were *C*. *pneumoniae*, *H*. *pylori* and *P*. *gingivalis*. For each analysis the forward primer sequence was fitted at the 5’-end with a 42–43 bp barcode sequence with unique recognition sequences allowing downstream deep sequencing of PCR products by 454 pyrosequencing. The reverse primer was tagged with a non-unique 31 bp sequence. Method optimization and validation was done using barcoded and tagged primers to ensure that the added sequences did not interfere with the specificity and sensitivity of the assay. The master mix contained 50 μL 2x Phusion Blood PCR Buffer (Thermo Fisher Scientific, Waltham, Massachusetts, USA) containing dNTPs, 38.5 μL DNAse/RNase and proteinase free molecular water (Sigma-Aldrich, St. Louis, Missouri, USA), 0.25 μM primer 357F-barcoded and 0.25 μM primer 1391R-tagged, 2 μL (2%) DMSO and 2 μL Phusion Blood II DNA polymerase (Thermo Fisher Scientific, Waltham, Massachusetts, USA). Total reaction volume was 100 μL, assuming a 5 μL volume for template in the form of angioplasty balloon, catheter control segment or 5 μL arterial blood. Amplification was performed on a Applied Biosystems Veriti^TM^ 96-Well Thermal Cycler (Thermo Fisher Scientific, Waltham, Massachusetts, USA) with 10 min initial denaturing at 98° C following 45 cycles of denaturation at 98° C for 5 sec, annealing at 63° C for 5 sec and extension at 72° C for 15 sec. Final extension was at 72° C for 1 min. Solid material in the PCR tube was pelleted by centrifugation at 1000g for 5 min and 10 μL of the supernatant was run on a 1% EDTA agarose gel for 30 min at 90V. The DNA ladder used was Thermo Scientific GeneRuler 1kb DNA Ladder (Thermo Fisher Scientific, Waltham, Massachusetts, USA). A positive signal was defined as a single visible band on the gel of the anticipated length.

Validation of the PCR assay was based on repeated experiments using dilution rows of human blood from healthy volunteers spiked with either *Pseudomonas aeruginosa* (PAO1) as model for Gram negative bacteria and *Streptococcus mutans* (UA159) as model for Gram positive bacteria. Dilution rows of blood with bacteria were prepared in 1.5 mL Eppendorf tubes. Sterile angioplasty balloons (MINI TREK^TM^ 2.00/20mm, Abbot Vascular, Costa Rica) were dipped into each Eppendorf tube and dilated for 5–10 sec, then deflated and cut into sterile RNAse/DNAas free tubes and frozen at -80° C for 24 h before analyses using the method described above. Repeated weighing of angioplasty balloons, before and after they were dipped in blood, revealed that the amount of blood able to adhere to the surface and in the folds of deflated angioplasty balloons varied between 1 and 5 μL. Therefore, 5 μL of arterial blood from each tube of the dilution rows were used as control for the angioplasty balloons. The bacterial concentration of the dilution rows was determined by spectrophotometry validated by cultivation and use of microscopic counting chambers.

## Results

### Validity of PCR assay

**Fig 1:**
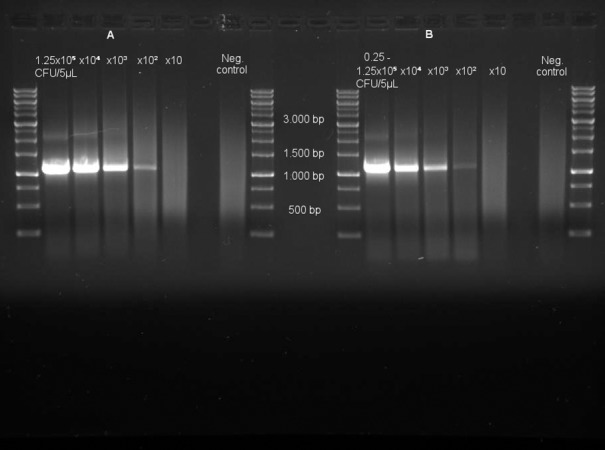
Photograph of agarose gel from one of the polymerase chain reaction (PCR) assay validation experiments. (A) Results from PCR performed on a 10-fold dilution row of 5 μL human blood from healthy volunteers spiked with *P*. *aeruginosa* in concentrations ranging from 1.25x10^5^-1.25x10 colony forming units (CFU)/5 μL. Detection limit was 125 CFU. (B) Results from PCR performed directly on angioplasty balloons dilated and deflated while dipped in the same 10-fold dilution row of blood with *P*. *aeruginosa*. Detection limit was 25–125 CFU depending on the amount of blood adhering to the balloon, which in repeated experiments varied from 1–5 μL. Blood samples without added bacteria served as negative controls.

Detection limits were between 25–125 colony-forming units (CFU) for angioplasty balloons dipped in blood spiked with a Gram negative bacterium (*P*. *aeruginosa*), depending on the amount of blood (1–5 μL) adhering to the angioplasty balloon. The detection limit was 125 CFU, when analysis was done directly on 5 μL spiked blood without angioplasty balloon (Fig 1). For Gram positive bacteria (*S*. *mutans*) the detection limit in repeated experiments was somewhat higher (10^2^−10^3^ CFU).

### Detection of DNA from oral microbiome

A total of 135 samples were analyzed. These included an angioplasty balloon, a catheter control segment, and an arterial blood sample from each of the 45 included patients. Patient dental history included 39.5% with periodontitis, 53.5% with partial or total edentulism, 4.7% with loose teeth, and 18.6% with bleeding when performing oral hygiene, with no apparent differences between subjects with stable and unstable coronary artery disease. Baseline characteristics of the study population are shown in Table 1.

**Table 1:** Baseline characteristics of study population.

	Stable angina (n = 15)	UAP/NSTEMI (n = 15)	STEMI (n = 15)	Total (n = 45)
**Age (mean±SD) years**	72.7±5.9	66.5±13.2	70.5±9.6	69.9±10.1
**Sex, women, n (%)**	3 (20%)	2 (13.3%)	4 (26.7%)	9 (20%)
**Diabetes**	5 (33.3%)	3 (20%)	2 (13.3%)	10 (22.2%)
**Hypertension**	13 (86.7%)	8 (53.3%)	10 (66.7%)	31 (68.9%)
**Hypercholesterolemia**	10 (66.7%)	12 (80%)	5 (33.3%)	27 (60%)
Smoking*	10 (66.7%)	8 (53.3%)	8 (53.3%)	26 (57.8%)
**Prior MI,**	1 (6.7%)	7 (46.7%)	2 (13.3%)	10 (22.2%)
**Family history of CAD**	3 (20%)	6 (40%)	4 (26.7%)	13 (28.9%)
**CRP ≤10/11-29/>30 mg/L, n**	12/3/0	11/4/0	10/5/0	33/12/0

CAD, coronary artery disease; UAP, unstable angina; MI, myocardial Infarction; NSTEMI, non ST-segment elevation MI; STEMI, ST-segment elevation MI; CRP, C-reactive protein.

*Active or prior smoking.

**Fig 2:**
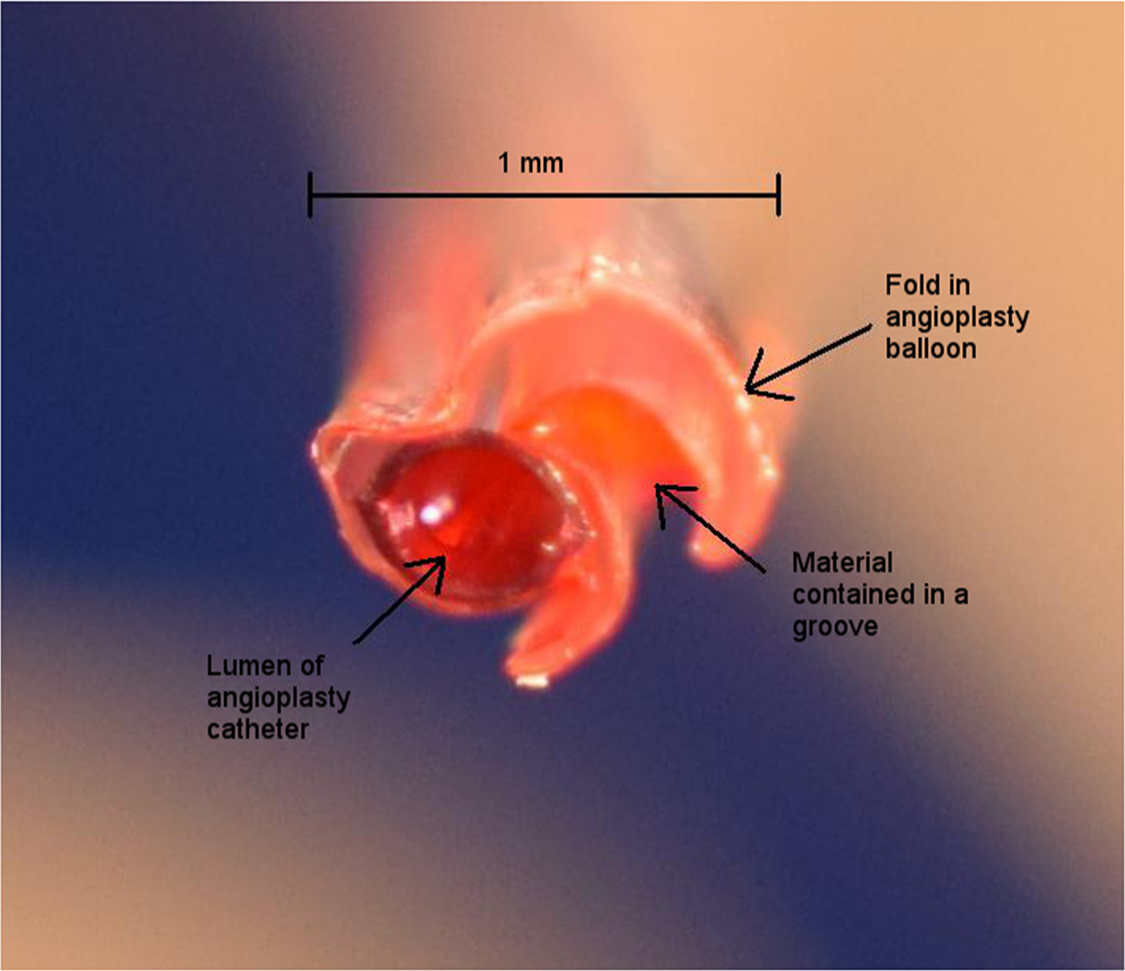
Transverse section of a deflated angioplasty balloon after percutaneous coronary intervention. Close-up photograph shows the folds and grooves that potentially may contain atherosclerotic tissue material from the site of coronary lesion dilatation.

The examined angioplasty balloons displayed folds and occasional grooves/pockets that potentially may have trapped atherosclerotic tissue material when balloons were deflated at the site of intracoronary high pressure dilatation (Fig 2).

**Fig 3:**
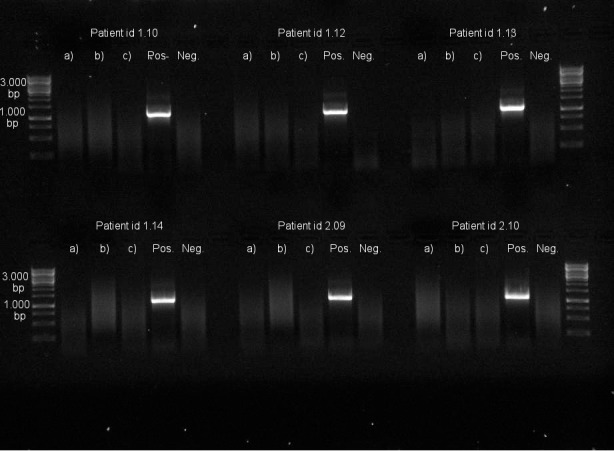
Photograph of agarose gel with polymerase chain reaction (PCR) results from six patient samples and controls. Patient id 1.10, 1.12, 1.13, and 1.14: stable angina; patient id 2.09 and 2.10: unstable angina/non ST-elevation myocardial infarction. Lane a) angioplasty balloon, lane b) catheter control segment, lane c) arterial blood. Positive controls were arterial blood spiked with 10^3^−10^4^ colony-forming units (CFU) *P*. *aeruginosa*. Negative controls were PCR master mix run without template.

None of the sampled angioplasty balloons or controls was found to be positive for detection of bacterial DNA. In all cases, the negative controls (PCR master mix without template) were negative, and all positive controls (5 μL of arterial blood spiked with 10^3^−10^4^ CFU *P*. *aeruginosa*) were positive (Fig 3). Since no samples were positive, no sample was selected for sequencing.

## Discussion

In this study, we developed a highly sensitive PCR-based assay that was capable of detecting 25–125 CFU and hence, theoretically, a similar number of single bacteria adhering to angioplasty balloons with optimized handling of samples by elimination of extraction and purification steps. However, we were unable to detect any bacterial DNA on angioplasty balloons or in control samples from 45 patients encompassing the full spectrum of symptomatic coronary artery disease (stable angina, UAP/NSTEMI, and STEMI) who underwent PCI. The periodontal status of the patients assessed by our standardized questionnaire suggested a prevalence of periodontitis of at least 39.5% which is comparable to other published data and clearly indicates that the negative results were not explained by absence of periodontal disease [13].

Several previous studies have reported on bacterial findings from endarterectomy samples from carotid or coronary arteries, and arteries removed at autopsy, but only very few studies have used coronary angioplasty balloons for sample retrieval and none of these studies reported results according to different manifestations of coronary artery disease (stable angina, UAP/NSTEMI or STEMI). With use of a ‘universal’ 16S RNA primer set, one of these studies reported an apparent 100% detection rate of bacterial DNA from 40 different species retrieved from coronary angioplasty balloons by use of an extraction, amplification, cloning and cultivation method from 18 patients with moderate to severe periodontitis selected from a total of 134 screened patients [35]. These investigators also found significant differences in the microbial diversity between angioplasty balloons and subgingival samples with *Firmicutes* being the most predominantly represented phylum from subgingival samples, and *Proteobacteria* dominating the findings from coronary angioplasty balloons. Indeed, many of the species that were isolated exclusively from angioplasty balloons are not known to be indigenous to the oral cavity although some species, including *P*. *gingivalis*, were isolated from both subgingival samples and coronary angioplasty balloons, which may be indicative of a possible translocation from the oral cavity [35]. However, it is important to note, that in contrast to the present study these patients were highly selected for presence of periodontitis, and control samples from arterial blood were not included.

Other investigators used a cloning technique and nested PCR with use of selective primers for detection of *C*. *pneumoniae* on washing medium from angioplasty balloons from 76 unselected patients with *de novo* coronary artery disease and found traces of this obligate intracellular bacterium in 39.5% of patients [36]. As controls, they used angioplasty balloons from 11 patients with coronary artery restenosis (the etiology of which is presumed to be different from that of native atherosclerosis with a predominant vascular smooth muscle cell proliferative response) and, interestingly, these were all negative [37]. In potential contrast to these results, however, results from another study in which the washing medium from coronary angioplasty balloons from 13 patients was analyzed with use of ‘universal’ 16S RNA primers and specific primers for *C*. *pneumoniae* and *CMV* were all negative [37]. To the best of our knowledge, our study is the first to report results with an optimized sampling process and internal validation of a highly sensitive PCR assay aimed at a broad spectrum of bacteria from the oral cavity which was then used on coronary angioplasty balloons and control samples from unselected patients with coronary artery disease clinically ranging from stable angina to STEMI. In contrast to previous studies, our assay allowed for the entire angioplasty balloon (or catheter control segment) to be used as direct template for PCR, thus minimizing the risk of contamination and loss of material through additional washing, extraction, and purification steps. However, despite that we demonstrated high sensitivity and specificity of our detection method, we did not find evidence of the presence of oral bacteria on coronary angioplasty balloons.

Compared with sample retrieval from coronary angioplasty balloons, catheter-based manual coronary thrombus aspiration during primary PCI in patients with STEMI has more recently provided another method for obtaining *in vivo* material from culprit coronary arteries for PCR analysis. Indeed, other researchers have previously examined aspirated intracoronary blood/thrombus from 81 unselected patients with STEMI with use of specific primers for five oral bacterial species and detected the presence of *Aggregatibacter actinomycetemcomitans*, *P*. *gingivalis*, and *Treponema denticola* in 17, 3, and 2 subjects, respectively [24]. Furthermore, a recent study analyzed aspirated thrombi from 101 unselected patients with STEMI and found 79 (78.2%) of thrombi positive, i.e., with significant increases of real-time PCR signals compared to the patient’s own arterial blood, for endodontic bacteria, e.g., *Streptococcus mitis* group, and 35 (34.5%) were positive for periodontal bacteria, e.g., *A*. *actinomycetemcomitans*, *P*. *gingivalis*, and *T*. *denticola* [25]. Interestingly, in a subgroup of these patients examined by panoramic tomography, a significant association was found between the presence of periapical abscesses and oral *S*. *mitis* and other viridans streptococci [25]. Although these results clearly suggest that thrombus aspiration may provide new mechanistic insights in this area of research, recent randomized trials have indicated that this procedure is not clinically useful for most patients with STEMI undergoing primary PCI, and such samples may therefore become less available in the future [45,46]. Importantly, it must be emphasized that detection of microbial DNA in samples from diseased coronary arteries does not represent proof of microbial viability within the atherosclerotic plaque, and even culturing of bacteria from coronary artery plaques is no proof of a causal relationship between microbial infection and atherosclerosis as, for example, these bacteria may merely be innocent bystanders compared to other mechanisms driving the complex atherosclerotic process [12,47].

In conclusion, with use of an optimized sampling process and internally validated sensitive PCR assay, we were not able to demonstrate the presence of bacterial DNA from the oral microbiome on angioplasty balloons from unselected patients representing the full spectrum of symptomatic coronary artery disease that underwent PCI and where a substantial proportion had a history of periodontitis. Although coronary angioplasty balloons are therefore unlikely to be useful for detection of bacteria with current PCR techniques in unselected patients with coronary artery disease, more studies are warranted to determine whether bacteria contribute to atherosclerosis and its clinical manifestations.
